# Retraction: progress of genome wide association study in domestic animals

**DOI:** 10.1186/2049-1891-4-3

**Published:** 2013-01-23

**Authors:** Hui Zhang, Zhipeng Wang, Shouzhi Wang, Hui Li

**Affiliations:** 1 Key Laboratory of Chicken Genetics and Breeding, Ministry of Agriculture, Harbin, 150030, People’s Republic of China; 2 College of Animal Science and Technology, Northeast Agricultural University, Harbin, 150030, People’s Republic of China

## Retraction

“Progress of genome wide association study in domestic animals” published in Journal of Animal Science and Biotechnology 2012 (3:26) [1], is regretfully retracted by the authors due to substantial textual overlap with previously published sources. We apologize to all affected parties for the inconvenience.
